# Adverse events are not increased by controlled delay in surgery of acute upper extremity fractures

**DOI:** 10.1038/s41598-023-28921-5

**Published:** 2023-02-02

**Authors:** Torkki Matias, Somersalo Axel, Kautiainen Hannu, Ax Maarit, Kiviranta Ilkka, Paloneva Juha

**Affiliations:** 1 Department of Surgery, Hospital Nova of Central Finland, Wellbeing Services County of Central Finland, Jyväskylä, Finland; 2 University of Helsinki, Haartmaninkatu 4, Helsinki, Finland; 3 Primary Health Care Unit, Kuopio University Hospital, Kuopio, Finland; 4 Folkhälsan Research Center, Helsinki, Finland; 5 Tampere University Hospital, Tampere, Finland; 6 University of Eastern Finland, Kuopio, Finland

**Keywords:** Trauma, Orthopaedics, Fracture repair, Health care economics, Public health

## Abstract

Management of the operative treatment of fractures is challenged by daily variation in patient flow. For upper limb fractures there has recently been an increasing tendency to temporarily discharge the patient to wait for a daytime operation to be performed during the next few days. The objective of this study was to study the safety of controlled delay in surgery. Upper limb fractures (N = 1 944) treated in a level 2 trauma center from 2010 to 2016 were identified and included in this retrospective cohort study with 5-year follow-up. Delay in surgery, inpatient time, readmissions, ED revisits and mortality were analyzed. Depending on the nature of the injury, controlled delay to surgery was implemented. Urgency of surgery was coded as green (2 days to 2 weeks) yellow (8–48 h) or red (less than 8 h). Harms and benefits to the groups were compared. Controlled delay of surgery (median delay of 5 days 7 h) was applied in 1 074 out of 1 944 fractures. The number of revisits to the emergency department (11.1% vs. 17.9–24.1%, p < 0.001) or hospital readmissions for any reason (0.99 per 100 person years in the delayed group vs. 3.74 and 4.35 in the non-delayed groups, p < 0.001) was no higher in patients with delayed (green) compared to those with non-delayed (yellow and red) operations. Inpatient time was significantly lower in the delayed group than other groups (IRR 2.31–3.36, (p < 0.001)). The standardized mortality ratio was 0.83 (CI 0.57–1.22) in the delayed group vs. 1.49 (CI 1.17–1.90) and 1.61 (CI 1.16–2.23) in the non-delayed groups. Controlled delay in upper limb trauma surgery did not lead to increased readmissions or mortality and was associated with reduced inpatient time.

## Introduction

Fractures of the upper limb comprise 59% of all fractures^1^. However, upper limb fractures account for only 28% of all fractures requiring inpatient care^2^. Increased mortality after these fractures has been documented^3^.

Traditionally, trauma patients requiring surgery have been treated as inpatients. During busy periods, time to operation could extend over several days, resulting in a high inpatient ward burden, decreased patient satisfaction, and excessive pressure on operating rooms^4^. Prioritizing systems have been developed to control for the uneven flow of patients requiring emergency surgery. The so-called traffic light system that has been developed to prioritize patients needing acute care and optimally allocate resources. Urgency of operation is coded based on clinical evaluation of the potential adverse effects of delay^5^. The code red indicates the most urgent type of emergency surgery, which should be performed within 8 h, and the code yellow less urgent surgery to be performed within 8–48 h^5^.

Recently, there has been a tendency to favor daytime surgery for upper extremity fractures. This has led to the development of a system of care called the “Green Line” in which the patient is temporarily discharged home from the emergency department or transferred to another care facility to await an operation to be performed during office hours within the next 2 weeks^4^. This system has been effective in controlling patient flow, especially in overcrowded conditions. However, delay in surgery is potentially harmful. The safety of delay in the surgical treatment of closed upper limb fractures has not been extensively studied. Hence, the aim of this study was to evaluate the potential harms, such as revisits to the emergency department, readmissions and mortality, and benefits, such as diminished inpatient time, caused by delay in these cases.

## Materials and methods

All patients operated for an upper limb fracture in the Central Finland Hospital (CFH) in Jyväskylä, Finland, between 1 January 2010 and 31 December 2016 were identified. The CFH, which is the only public hospital in the Central Finland Healthcare District, offers trauma care to a population of 254,000, which is approximately 5% of the population of Finland. Patients under age 16 were excluded from the study.

The research data were retrieved from an electronic medical record (EMR) system (Effica, Tieto Corporation, Helsinki, Finland). Patient mortality and educational status at the end of 2017 were obtained from Statistics Finland. This timepoint marked the end of the follow-up. Patient data in Statistics Finland is patient-specific and matched to patients by their Finnish social security code. The electronic medical record data included patient age, inpatient treatment, time to operation from the decision to operate, urgency of surgery, diagnostic code (ICD-10) and procedure code (NOMESCO, Finnish version) to identify operated patients, the ASA (American Society of Anesthesiologists) classification, and significant comorbidities (diabetes mellitus, musculoskeletal diseases, neurological diseases, cardiovascular diseases and respiratory diseases).

The exact time of the decision to perform surgery was only available after 1.1. 2013, and thus delay in the start of surgery was analyzed only for patients operated during 2013–2016. Patients operated during 2010–2013 were included in all the other analyses.

Surgical urgency was determined simultaneously with the decision for operative treatment by the surgeon on call in the emergency department. Patients were divided into 3 urgency groups (red: 0–8 h, yellow: 8–48 h and green: 2–14 days) according to fracture type (for example, open fractures were coded red), comorbidities, other injuries, and factors affecting survival in the patient’s normal living conditions such as ability to take care of daily hygiene and eating. Patients who needed inpatient treatment, for example due to complicated pain medication, but did not have other reasons for needing more urgent procedures were coded yellow. For patients able to return home to wait for the operation with sufficient pain medication, and for whom fracture immobilization was available and who had no associated injuries, were usually coded green^4^.

If the patient had been operated multiple times during the treatment period, the first operation was chosen for analysis in this study. Readmissions for any reasons and visits to the emergency department without readmission during the first 3 months after the first treatment period were identified. Primary outcome criteria were mortality, length of stay, readmission and revisits.

### Statistics

Data are presented as means with standard deviation (SD), median with interquartile range (IQR) or as counts with percentages. Statistical comparisons between the 3 urgency groups in study population characteristics were performed using analysis of variance (ANOVA) for continuous variables and Pearson's chi-square for categorical variables. The number of inpatient days and readmission incidence rate were calculated using Poisson regression models. Poisson regression was tested using the goodness-of-fit of the model, and the assumption of overdispersion in the Poisson model was tested using the Lagrange multiplier test. Time-to-event analysis was based on the Kaplan–Meier failure function. The Cox proportional hazards model was used to calculate the adjusted hazard ratios (HR). The normality of variables was evaluated using the Shapiro–Wilk W test. All analyses were performed using Stata version 16.0 (Stata Corp., College Station, TX).

The standardized mortality ratio (SMR) was calculated as the ratio between the number of deaths observed and the number of deaths expected, with 95% confidence intervals (CIs), assuming a Poisson distribution. The expected number of deaths was calculated on the basis of age-, sex- and calendar period-specific mortality rates in the Finnish population using data obtained from Statistics Finland.

### Ethical approval

Institutional review board of Central Finland Health care District approved the study on December 16, 2012. The authors confirm that all research was performed in accordance with relevant regulations. No external funding obtained.

### Informed consent

N/A, informed consent was not required to conduct this register study. The study subjects were not contacted.

## Results

A total of 1944 upper limb fractures were identified. Of these, 55.2% (n = 1074; 574 women), were coded green and thus treated within 2 days to 2 weeks, 30.6% (n = 594; 357 women) were coded yellow (urgency 8–48 h), and 14.2% (n = 279; 234 women) were coded red (urgency less than 8 h) (Table 1).

**Table 1:** **C**haracteristics of the study population.

Urgency of operation*	0–8 h (red) N = 276	8–48 h (yellow) N = 594	2–14 days (green) N = 1074	p value
Age, mean (SD)	56 (20)	60 (20)	50 (18)	< 0.001
Women, n (%)	134 (48.6)	357 (60.1)	574 (53.4)	0.003
Education years, mean (SD)	10.9 (2.9)	10.8 (3.2)	12.0 (2.9)	< 0.001
ASA, n (%)				< 0.001
1	63 (26.8)	124 (23.9)	427 (45.0)	
2	83 (35.3)	170 (32.8)	345 (36.4)	
3	80 (34.0)	196 (37.8)	164 (17.3)	
4	9 (3.8)	29 (5.6)	12 (1.3)	
Fracture location**, n (%)				< 0.001
Distal forearm	67 (24.3)	152 (25.6)	435 (40.5)	
Proximal humerus	52 (18.8)	142 (23.9)	93 (8.7)	
Proximal forearm	35 (12.7)	88 (14.8)	78 (7.3)	
Clavicle	26 (9.4)	28 (4.7)	91 (8.5)	
Finger phalanx	31 (11.2)	58 (9.8)	180 (16.8)	
Distal humerus	39 (14.1)	91 (15.3)	40 (3.7)	
Shaft of forearm	27 (9.8)	37 (6.2)	37 (3.4)	
Metacarpal	16 (5.8)	20 (3.4)	109 (10.1)	
Humerus diaphysis	12 (4.3)	31 (5.2)	25 (2.3)	
Carpal	2 (0.7)	4 (0.7)	28 (2.6)	
Scapulae	6 (2.2)	8 (1.3)	8 (0.7)	
Comorbidies**, n (%)				
Cardiovascular	121 (43.8)	268 (45.1)	292 (27.2)	< 0.001
Neurological	52 (18.8)	110 (18.5)	154 (14.3)	< 0.001
Respiratory	52 (18.8)	110 (18.5)	154 (14.3)	0.039
Musculosceletal	52 (18.8)	123 (20.7)	138 (12.8)	< 0.001
Diabetes mellitus	43 (15.6)	84 (14.1)	71 (6.6)	< 0.001

*According to the traffic light coding. **Number and percentage calculated for each group.

Clarification: the delay from the treatment decision to surgery has only been available since 2013. Before 2013, 788 (181 red, 260 yellow, and 347 green) patients were operated.

The fracture sites most commonly coded green were the distal forearm (40.5% in the subgroup), finger phalanx (16.8%), metacarpal (10.1%) and proximal humerus (8.7%); those most commonly coded yellow were the distal forearm (25.6% in the subgroup), proximal humerus (23.9%), distal humerus (15.3%) and proximal forearm (14.8%); and those most commonly coded red were the distal forearm (24.3% in the subgroup), proximal humerus (18.8%), distal humerus (14.1%) and proximal forearm (12.7%) (Table 1).

In addition to the 1944 operated fractures, the operations planned for 75 (3.7%) patients with an upper limb fracture coded green were cancelled. The need for surgery had been re-evaluated in the majority of these cases in favor of non-operative treatment. Patient death was not a reason for cancellation in any of these cases. As they were not operated, these patients were not included in the study analyses.

In green group, median delay from the treatment decision to surgery start was 127 h (5 days 7 h) (IQR 71.6, 169.5). In yellow group, median delay was 20.2 h (IQR 12.5, 32.5). In red group, the median delay was 5.8 h (IQR 3.0, 20.7). All operations were not performed during the desired time frame due to uneven patient flow and limited operating room resources during out-of-office hours. In the green coded group, 98% of operations were performed within the original 14-day time frame, and in the yellow coded group 89% of operations were performed within 48 h. In the red-coded group, however, the 8-h goal was achieved in only 54% of cases. Delay time from the treatment decision to surgery has only been available since 2013. Before 2013, operations were performed on 788 (181 red, 260 yellow and 347 green) patients.

**Figure 1:**
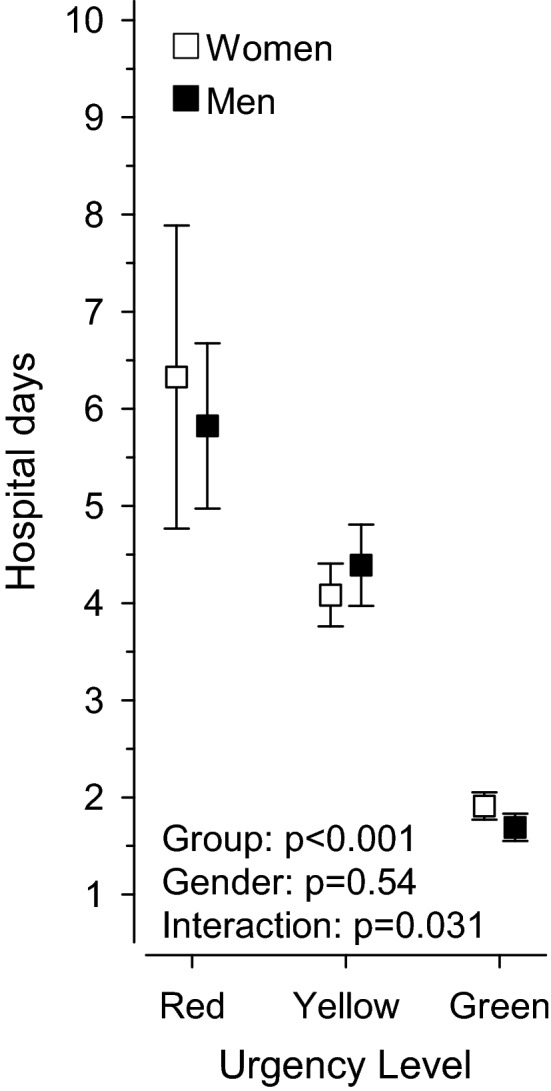
Mean length of hospital stay (days). Urgency of emergency operation: red = 0–8 h; yellow = 8–48 h; green = 2–14 days. Whiskers represent 95% confidence intervals.

Mean inpatient time was 3.2 days for all patients with a fracture of the upper limb. For the green coded patients, mean inpatient time was 1.8 (SD 1.5) days. For the yellow coded patients, mean inpatient time for a fracture in the corresponding bone area was 4.3 (SD 2.7) days (IRR 2.31 compared to green group) and for the red coded patients mean inpatient time was 6.2 (SD 6.9) days (IRR 3.36 compared to green group) (p < 0.001) (Fig. 1).

The mean ASA score was statistically significantly lower in the green group (1.75 (median 2)) compared to the yellow [2.25 (median 2)] and red [2.15 (median 2)] groups. Moreover, fewer comorbidities (cardiovascular diseases, neurological diseases, respiratory diseases, musculoskeletal diseases and diabetes) were observed in the green than the other groups (Table 1).

The number of readmissions for any reason during the first 3 months was 32 (3.0%) in the green group and 66 (11.1%) and 36 (12.9%) in the yellow and red group, respectively. Readmission incidence was 0.99 (CI 0.68–1.40) per 100 person years in the green group, 3.74 (CI 2.89–4.76) per 100 person years in the yellow group, and 4.35 (CI 3.05–6.02) per 100 years in the red group (p for linearity p < 0.001). The most common diagnoses for readmission were cardiovascular diseases (20.1%) followed by diseases of the digestive system (9.7%) and symptoms, signs and abnormal clinical and laboratory findings not elsewhere classified (9.7%) (Table 2).

**Table 2:** Causes of readmission within 30 days.

ICD-10	Explanation	Urgency of operation			N, total
		0–8 h (N, red code)	8–48 h (N, yellow code)	2–14 days (N, green code)	
A	Certain infectious and parasitic diseases	4	7	1	12
D	Benign neoplasms or diseases of the blood and blood-forming organs	1	2	0	3
E	Endocrine, nutritional and metabolic diseases	0	1	1	2
F	Mental, behavioral and neurodevelopmental disorders	0	1	0	1
G	Diseases of the nervous system	3	4	0	7
H	Diseases of the eye and adnexa	3	3	3	9
I	Diseases of the circulatory system	7	17	3	27
J	Diseases of the respiratory system	0	4	1	5
K	Diseases of the digestive system	1	6	6	13
L	Diseases of the skin and subcutaneous tissue	1	1	1	3
M	Diseases of the musculoskeletal system and connective tissue	0	4	2	6
N	Diseases of the genitourinary system	2	3	1	6
O	Pregnancy, childbirth and the puerperium	0	1	1	2
R	Symptoms, signs and abnormal clinical and laboratory findings, not elsewhere classified	5	6	2	13
S	Injuries	5	1	1	7
T	Injury, poisoning and certain other consequences of external causes	3	4	4	11
Z	Factors influencing health status and contact with health services	1	1	1	3
UK	Unknown	0	0	4	4

The number of visits to the emergency department during the 3 months after the end of the treatment period was 119 (11.1%) in the green group, 143 (24.1%) in the yellow group and 50 (17.9%) in the red group. The incidence of emergency department visits was 3.70 (CI 3.06–4.43) per 100 person years in the green group, 8.11 (CI 6.83–9.55) per 100 person years in the yellow and 6.04 (CI 4.48–7.96) per 100 person years in the red group (p for linearity p < 0.001). The most common reasons for visiting the emergency department were injuries (41.7%) and other consequences of external causes (15.5%) (Table 3).

**Table 3:** Causes of postoperative visits in emergency department within 30 days.

ICD-10	Explanation	Urgency of operation			N, total
		0–8 h (N, red code)	8–48 h (N, yellow code)	2–14 days (N, green code)	
AB	Certain infectious and parasitic diseases	4	4	1	9
C	Neoplasms	0	1	0	1
D	Benign neoplasms or diseases of the blood and blood-forming organs	0	2	1	3
E	Endocrine, nutritional and metabolic diseases	0	2	1	3
G	Diseases of the nervous system	1	8	0	9
I	Diseases of the circulatory system	5	25	3	33
J	Diseases of the respiratory system	1	7	3	11
K	Diseases of the digestive system	1	3	7	11
L	Diseases of the skin and subcutaneous tissue	0	2	0	2
M	Diseases of the musculoskeletal system and connective tissue	3	6	6	15
N	Diseases of the genitourinary system	1	2	0	3
R	Symptoms, signs and abnormal clinical and laboratory findings, not elsewhere classified	10	18	8	36
S	Injury	21	59	65	145
T	Injury, poisoning and certain other consequences of external causes	10	17	27	54
VXY	External causes of morbidity	0	2	3	5
Z	Factors influencing health status and contact with health services	1	4	3	8

**Figure 2:**
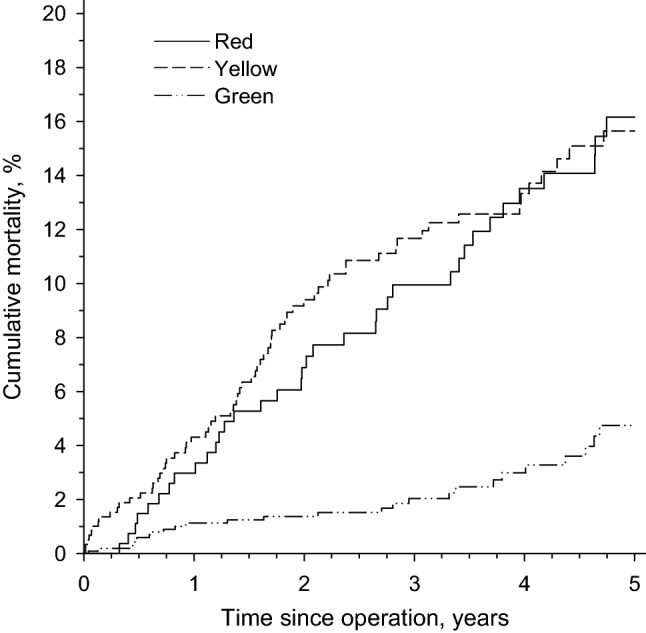
Cumulative mortality. Urgency of emergency operation: red = 0–8; yellow = 8–48 h; green = 2–14 days.

Of all the patients treated for upper limb fractures, 129 patients (87 women) died during the 5-year follow-up. Kaplan–Meyer cumulative mortality was 16.8% (CI 12.8–22.0%) for all patients: 7.3% (CI 5.3–10.0%) for men and 12.9% (CI 10.4–16.0%) for women. Age- and sex-adjusted hazard ratios for 5- year mortality were 2.60 (CI 1.55–4.36) when comparing the red and green groups and 3.50 (CI 1.57–3.99) when comparing the yellow and green groups (Fig. 2).

30-day mortality was 0.1% (CI 0.0–0.7%) in the green group, 0.9% (CI 0.04–2.0%) in the yellow group and 0.0% (one-sided upper 97.5% CI 1.3%) in the red group. 90-day mortality was 1.9% (CI 0.1–0.8%) in the green group, 1.5 (CI 0.8–2.9%) in the yellow group and 0.0% (one-sided upper 97.5% CI 2.6%) in the red group.

No statistically significant differences in the causes of death were observed between the groups. The most common cause of death was cardiovascular diseases (42%) followed by neoplasms (18%) (Table 4).

**Table 4:** Causes of death during the follow-up period.

ICD-10	Explanation	Urgency of operation		
		0–8 h (red) N = 36 (%)	8–48 h (yellow) N = 67 (%)	2–14 days (green) N = 26 (%)
A	Certain infectious and parasitic diseases	0 (0)	2 (3)	0 (0)
C	Neoplasms	6 (17)	10 (15)	7 (27)
E	Endocrine, nutritional and metabolic diseases	0 (0)	0 (0)	2 (8)
F	Mental, behavioral and neurodevelopmental disorders	1 (3)	5 (7)	0 (0)
G	Diseases of the nervous system	5 (14)	11 (16)	3 (12)
I	Diseases of the circulatory system	20 (56)	25 (37)	9 (35)
J	Diseases of the respiratory system	1 (3)	1 (1)	1 (4)
K	Diseases of the digestive system	1 (3)	4 (6)	4 (15)
M	Diseases of the musculoskeletal system and connective tissue	1 (3)	0 (0)	0 (0)
VXY	External causes of morbidity	1 (3)	9 (13)	0 (0)

The standardized mortality ratio (SMR) was 1.61 (CI 1.16–2.23) in the red group, 1.49 (CI 1.17–1.90) in the yellow group and 0.83 (CI 0.57–1.22) in the green group. The difference between the green and red groups (p = 0.011) and between the green and yellow groups (p = 0.012) was statistically significant.

## Discussion

In this study, we analyzed the safety of controlled delay in the operative treatment of patients with upper limb fractures. We found that mortality among upper extremity fracture patients with a delayed operation (2–14 days) was no greater than among those with a more rapidly implemented operation (0–48 h). Delaying the operation did not increase the numbers of revisits to the emergency department or hospital readmissions for any reasons. Moreover, inpatient time in the hospital ward was significantly lower in the delayed group than other groups. This decrease in inpatient time was expected, as patients whose operative treatment is delayed are healthier, are likely to have less severe injuries and do not wait for their operation as inpatients but are discharged home to wait for their operation after the decision to opt for operative treatment has been taken^4^.

Increased mortality after upper limb fractures has been reported. In a study on patients from the same hospital district as in our study (using data from the years 2002 to 2008), the SMR was 1.5 for inpatients with upper limb fractures^3^. Significantly increased mortality after proximal humeral but not wrist fractures has also been reported in the elderly population^6,7^. Fractures in younger patients seem to be associated with a higher increase in relative mortality than fractures in older patients^6,8,9^. In our study, the patients in the green group were a little younger than those in the red or yellow groups and thus, according to previous findings, should have shown higher relative mortality; however, this was not the case. The SMR was 0.83 (CI 0.57–1.22) in the green group, suggesting a trend toward lower mortality than in the general population. However, this finding was not statistically significant. The low SMR in the green group is likely a result of patient selection of generally healthy persons in this group. Admittedly, the proportion of wrist fractures, which would not be expected to influence mortality^6^ was higher in the green group (41%) than in the red (24%) or yellow (26%) groups. In this study, we did not predict the influence of delay on mortality but showed that mortality was not higher in patients with longer waiting period to surgery. For wrist fractures, surgical delay does not seem to have a significant effect on the results of the treatment^10^. If the operative treatment for wrist fractures is delayed due to secondary displacement, it may lead to poorer functional outcomes^11^. However, there is no evidence that planned, controlled delay without secondary displacement has the same effect on functional outcomes. Similarly, with clavicle fractures, no statistically significant difference in healing has been reported between early or delayed (up to 12 weeks) fixation^12^, and thus these fractures seem well suited to our controlled, delayed method of surgical management. To our knowledge, no studies have reported the effects of delaying surgery in operative elbow fractures in adults. In proximal humeral fractures, a delay of more than 5 days in surgery was associated with increased loss of fixation rates^13^. Moreover, contrary to our study, a delay of 3 days compared to a shorter delay in treating proximal humeral fractures increased inpatient morbidity and postoperative length of stay. However, the delay in surgery did not increase the risk of death after controlling for comorbidities and age^14^.

There are many benefits for patients if operative treatment for an upper limb trauma can be scheduled over the next few post-trauma days. Such patients are temporarily discharged, instead of waiting, and sometimes fasting, for up to several days as inpatients to wait for their operation during busy periods. It also allows hospitals to better cope with trauma patients in greater need of acute care and schedule surgery for non-acute cases during office hours when resources are fully available. Releasing resources from non-daytime activities also benefits trauma patients requiring more rapid acute care (e.g., hip fracture patients). In addition, compared to the traditional treatment model where patients are directly transferred to the ward from the emergency department to await surgery, the shorter inpatient time resulting from temporally discharged patients frees ward capacity. It should be noted that not all fractures are suitable for delayed surgery. The influence of delayed surgery, especially with respect to hip fractures, on mortality has previously been studied. In hip fractures, early surgery, i.e., less than 48 h after injury, decreased late mortality compared to delayed surgery^15^. Upper limb fractures do not compromise a patient’s mobility and therefore it is easier for these patients to wait at home for an operation. However, patients needing extensive immobilization, advanced pain management, or active follow up of vital functions, or who have many preliminary comorbidities or other conditions warranting impatient care are typically admitted to await surgery.

The present study is a register study, meaning that patients were assigned to different urgency groups based on the anatomic location of the fracture, additional injuries, and patient characteristics according to current local clinical guidelines instead of randomization. The groups also showed demographic differences. A strength in this study is the comprehensiveness of our hospital in the treating of fracture patients in its catchment area. Moreover, it was possible to calculate a standardized mortality ratio comparing our patients with the Finnish general population. A limitation of the study is that the results cannot be applied to the population under 16 years of age, as this segment was excluded from our analysis. However, there are no obvious reasons why controlled delay in care could not also be applied in this young population. A further limitation is that our register data did not allow us to analyze all the potential risks of surgical delay or functional results of treatment. Another limitation is that our groups were not adjusted and thus there was some heterogeneity between the group characteristics which will induce some bias in the results. Uneven patient flow and limited operating room resources during out-of-office hours also impact patient waiting times. In this study, a shift towards the conservative treatment of proximal humeral fractures during the study period limits the generalizability of results. Moreover, the inclusion in the study of multitrauma patients lenghtens the mean inpatient time in the red and yellow groups.

The effect of delay in surgery for upper limb fractures would be best established using a RCT. However, in the present clinical setting, such a study is unlikely for two main reasons. First, our controlled delay system enables efficient trauma treatment with minimal inpatient time and good patient satisfaction, and second, because this setting is already in use, it would be difficult to get real case control setting in a retrospective study design. Future research could analyze the relationship between delay in surgery and functional outcomes. The socioeconomic impacts of the controlled delay system also merit investigation.

## Conclusion

We found no increase in mortality among patients treated operatively with a controlled delay of less than 2 weeks. In addition, length of hospital stay and need for inpatient treatment decreased when patients were discharged home pending the setting of a date for their operation. Postoperative revisits to the emergency department for any reason after surgery were also less common in these patients than in those admitted to hospital for more urgent surgery.

## Data Availability

The dataset supporting the conclusions of this article are available from the corresponding author on reasonable request.
